# Hypermucoviscous Carbapenem-Resistant *Klebsiella pneumoniae* ST25 Infect Human Intestinal Epithelial Cells and Induce Moderate Inflammation

**DOI:** 10.3390/ijms24108804

**Published:** 2023-05-15

**Authors:** Stefania Dentice Maidana, Mariano Elean, Kohtaro Fukuyama, Yoshiya Imamura, Leonardo Albarracín, Sudeb Saha, Yoshihito Suda, Shoichiro Kurata, María Ángela Jure, Haruki Kitazawa, Julio Villena

**Affiliations:** 1 Laboratory of Immunobiotechnology, Reference Centre for Lactobacilli (CERELA-CONICET), Tucuman 4000, Argentina; stefi.dentice@gmail.com (S.D.M.); melean@cerela.org.ar (M.E.); lalbarracin@herrera.unt.edu.ar (L.A.); 2 Laboratory of Antimicrobials, Institute of Microbiology “Luis C. Verna”, Faculty of Biochemistry, Chemistry and Pharmacy, National University of Tucuman, Tucuman 4000, Argentina; magejure@gmail.com; 3 Food and Feed Immunology Group, Laboratory of Animal Food Function, Graduate School of Agricultural Science, Tohoku University, Sendai 980-8577, Japan; kotaro.fukuyama.p8@dc.tohoku.ac.jp (K.F.); yoshiya.imamura.p8@dc.tohoku.ac.jp (Y.I.); sudeb.saha.a8@tohoku.ac.jp (S.S.); 4 Livestock Immunology Unit, International Education and Research Centre for Food and Agricultural Immunology (CFAI), Graduate School of Agricultural Science, Tohoku University, Sendai 980-8577, Japan; 5 Department of Dairy Science, Faculty of Veterinary, Animal and Biomedical Sciences, Sylhet Agricultural University, Sylhet 3100, Bangladesh; 6 Department of Food, Agriculture and Environment, Miyagi University, Sendai 980-8572, Japan; suda@myu.ac.jp; 7 Laboratory of Molecular Genetics, Graduate School of Pharmaceutical Sciences, Tohoku University, Sendai 980-8578, Japan; shoichiro.kurata.d5@tohoku.ac.jp

**Keywords:** *Klebsiella pneumoniae*, carbapenem resistant, intestinal infection, genomic, sequence type 25

## Abstract

*Klebsiella pneumoniae* is an opportunistic pathogen that can produce moderate and severe infections in immunosuppressed hosts. In recent years, an increase in the isolation of hypermucoviscous carbapenem-resistant *K. pneumoniae* with sequence type 25 (ST25) in hospitals in Norwest Argentina was observed. This work aimed to study the virulence and inflammatory potential of two *K. pneumoniae* ST25 strains (LABACER01 and LABACER27) in the intestinal mucosa. The human intestinal Caco-2 cells were infected with the *K. pneumoniae* ST25 strains, and their adhesion and invasion rates and changes in the expression of tight junction and inflammatory factors genes were evaluated. ST25 strains were able to adhere and invade Caco-2 cells, reducing their viability. Furthermore, both strains reduced the expression of tight junction proteins (occludin, ZO-1, and claudin-5), altered permeability, and increased the expression of TGF-β and TLL1 and the inflammatory factors (COX-2, iNOS, MCP-1, IL-6, IL-8, and TNF-α) in Caco-2 cells. The inflammatory response induced by LABACER01 and LABACER27 was significantly lower than the one produced by LPS or other intestinal pathogens, including *K. pneumoniae* NTUH-K2044. No differences in virulence and inflammatory potential were found between LABACER01 and LABACER27. In line with these findings, no major differences between the strains were found when the comparative genomic analysis of virulence factors associated with intestinal infection/colonization was performed. This work is the first to demonstrate that hypermucoviscous carbapenem-resistant *K. pneumoniae* ST25 infects human intestinal epithelial cells and induces moderate inflammation.

## 1. Introduction

*Klebsiella pneumoniae* is a Gram-negative, encapsulated, non-motile, and facultative anaerobe bacteria, which is normally present in the environment and a commensal in the gastrointestinal tracts of humans and animals [1]. In recent years, *K. pneumoniae* has gained more attention globally due to the increasing number of severe infections and the rising scarcity of effective treatments [2]. The new hypervirulent (hypermucoviscous) *K. pneumoniae* variants are becoming a public health concern due to the severity of infections, including pneumonia, bacteremia, pyogenic liver abscess, and urinary tract infections, endangering the lives of patients [3]. The prevalence of hypervirulent *K. pneumoniae* infections rate ranges from 5% to 35% in Western countries, whereas in Asian countries rate ranges from 18% to 87% [4,5]. Of note, there is an increase in the carbapenem-resistant *K. pneumoniae* isolates in Asia (1.53%), USA (16.05%), and South America (21.67%) [6]. Thus, the rapid dissemination of this pathogen is becoming a major threat to public health worldwide.

As an opportunistic pathogen, *K. pneumoniae* can produce moderate and severe infections in immunosuppressed hosts. Recent studies have shown that most *K. pneumoniae* infections begin in the intestinal epithelium, from which they translocate and cause infections in other parts of the body [7,8]. The virulence and pathogeny of *K. pneumoniae* strains have been associated with the presence of virulence factors, such as the mucoviscosity associated protein gene A (*magA*) [9,10], capsule [11], fimbriae [11], *rmpA* gene [12,13], outer membrane proteins [11,14], and type 6 secretion system (T6SS) [11,15], and siderophores for iron uptake [11]. Studies in animal models and Caco-2 cells have made significant advances in the understanding of the mechanisms involved in *K. pneumoniae* intestinal colonization and its translocation to other host tissues. It was shown that Rho GTPase- and phosphatidylinositol 3-kinase/Akt pathway are involved in the translocation of this pathogen in Caco-2 cells [16]. Furthermore, transcriptome studies in Caco-2 cells challenged with *K. pneumoniae* found significant up-regulation of genes of the TGF-β signaling pathway and the Tolloid-like protein TLL1 [8], indicating the role of this pathway in adhesion to and invasion of intestinal epithelial cells. Of note, several *K. pneumoniae* clinical isolates were evaluated in these works, and it was found that it has a strain dependent ability to adhere and invade Caco-2 cells [8,16]. This fact emphasizes the importance of evaluating each clinically important isolate for its ability to colonize and invade the intestinal mucosa.

Our previous studies revealed that hypervirulent carbapenem-resistant *K. pneumoniae* from the sequence type 25 (ST25), which is frequently isolated from clinical samples in Argentina currently, can cause a wide variety of infectious diseases, including respiratory and urinary tract infections [17,18,19]. As the virulence of these bacteria and the demographic characteristics of the patients they infect begin to shift, understanding how *K. pneumoniae* is transmitted, and the factors related to their pathogenicity is important for treating patients. There are few studies characterizing the virulence and inflammatory activities of *K. pneumoniae* ST25 in the respiratory tract [17,19,20]; however, their virulence and inflammatory potential in the intestinal mucosa was not evaluated before. Thus, the aim of this study was to explore the infective capacity of *K. pneumoniae* ST25 strains and the inflammatory responses in human colorectal Caco-2 cells. For this purpose, two multidrug-resistant intrahospital *K. pneumoniae* ST25 strains (LABACER01 and LABACER27) with different virulence patterns were considered. In addition, a comparative genomic analysis was performed to characterize the virulence factors specifically associated with intestinal infection.

## 2. Results

### 2.1. K. pneumoniae ST25 Adhere and Infect Caco-2 Cells

**Figure 1:**
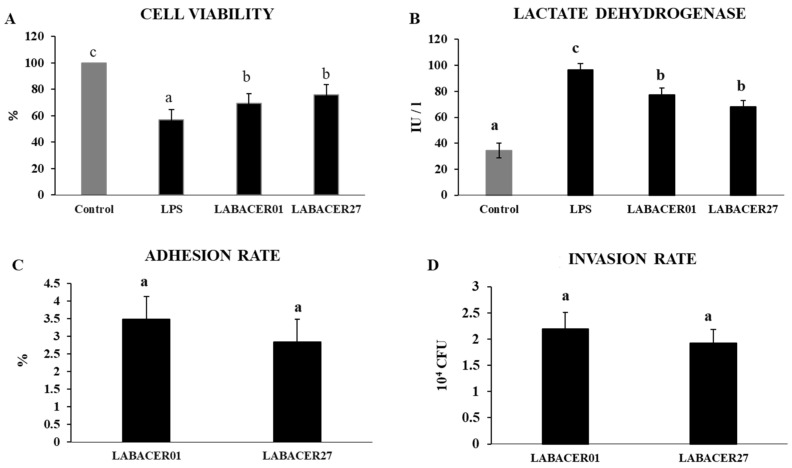
Cell viability (**A**), lactate dehydrogenase (LDH) release (**B**), adhesion rate (**C**), and invasion rate (**D**) in human colorectal epithelial Caco-2 cells challenged with KPC-2-producing hypermucoviscous ST25 strains of *Klebsiella pneumoniae*. Caco-2 cells were challenged with *K. pneumoniae* LABACER01, *K. pneumoniae* LABACER27, or LPS for 24 h. Untreated cells were used as controls. Results represent data from three independent experiments. Letters indicate significant differences between the groups, a < b < c, *p* < 0.05.

We first evaluated the capacity of *K. pneumoniae* ST25 LABACER01 and LABACER27 strains to adhere to and infect human intestinal epithelial cells. The challenge of Caco-2 cells with *K. pneumoniae* ST25 for 24 h significantly reduced viability and increased lactate dehydrogenase (LDH) release than unchallenged control cells, indicating cellular toxicity (Figure 1A,B). No differences were found when both parameters were compared in cells challenged with LABACER01 and LABACER27. Of note, the effect of both *K. pneumoniae* ST25 strains was significantly lower than that induced by the LPS challenge (Figure 1A,B). The results also demonstrated the capacity of LABACER01 and LABACER27 to adhere (Figure 1C) and invade (Figure 1D) Caco-2 cells. No significant differences were found between the two isolates when adhesion and invasion were compared.

### 2.2. K. pneumoniae ST25 Infection Affects Tight Junction Proteins Expression in Caco-2 Cells 

**Figure 2:**
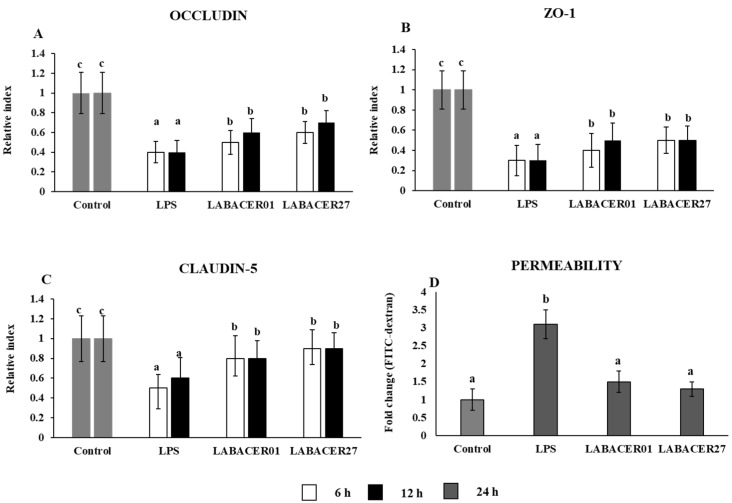
Relative expression of tight junction protein (**A**) occludin, (**B**) ZO-1, and (**C**) claudin-5 in human colorectal epithelial Caco-2 cells challenged with KPC-2-producing hypermucoviscous ST25 strains of *Klebsiella pneumoniae*. Caco-2 cells were challenged with *K. pneumoniae* LABACER01, *K. pneumoniae* LABACER27, or LPS for 6 or 12 h. Untreated cells were used as controls. (**D**) Caco-2 cell large solute permeability by using the FITC-dextran flux method after the stimulation of cells with *K. pneumoniae* LABACER01, *K. pneumoniae* LABACER27, or LPS for 24 h. Results represent data from three independent experiments. Letters indicate significant differences between the groups, a < b < c, *p* < 0.05.

To investigate the effect of *K. pneumoniae* ST25 on the intestinal barrier function, we evaluated the relative expression of occludin, ZO-1, and claudin-5 on Caco-2 cells challenged with *K. pneumoniae* LABACER01, LABACER27, or stimulated with LPS at different time points (6 and 12 h). As shown in Figure 2, Caco-2 cells exhibited a significant downregulation of gene expression for occludin, ZO-1, and claudin after *K. pneumoniae* ST25 or LPS stimulation, compared to the unchallenged control group. However, the gene expression of occludin, ZO-1, and claudin was higher in the LABACER01 and LABACER27 groups compared with the cells stimulated with LPS at both time points (Figure 2). We also measured the permeability of the Caco-2 cells monolayer by using the FITC-dextran method. As shown in Figure 2, the levels of FITC-dextran in the basal compartment of cell cultures were significantly increased in the group treated with LPS when compared to the controls. In contrast, the concentrations of translocated FITC-dextran in cells challenged with the *K. pneumoniae* ST25 were not different from control and significantly lower compared to cells stimulated with LPS.

### 2.3. K. pneumoniae ST25 Infection Affect COX-2 and iNOS Expression in Caco-2 Cells

**Figure 3:**
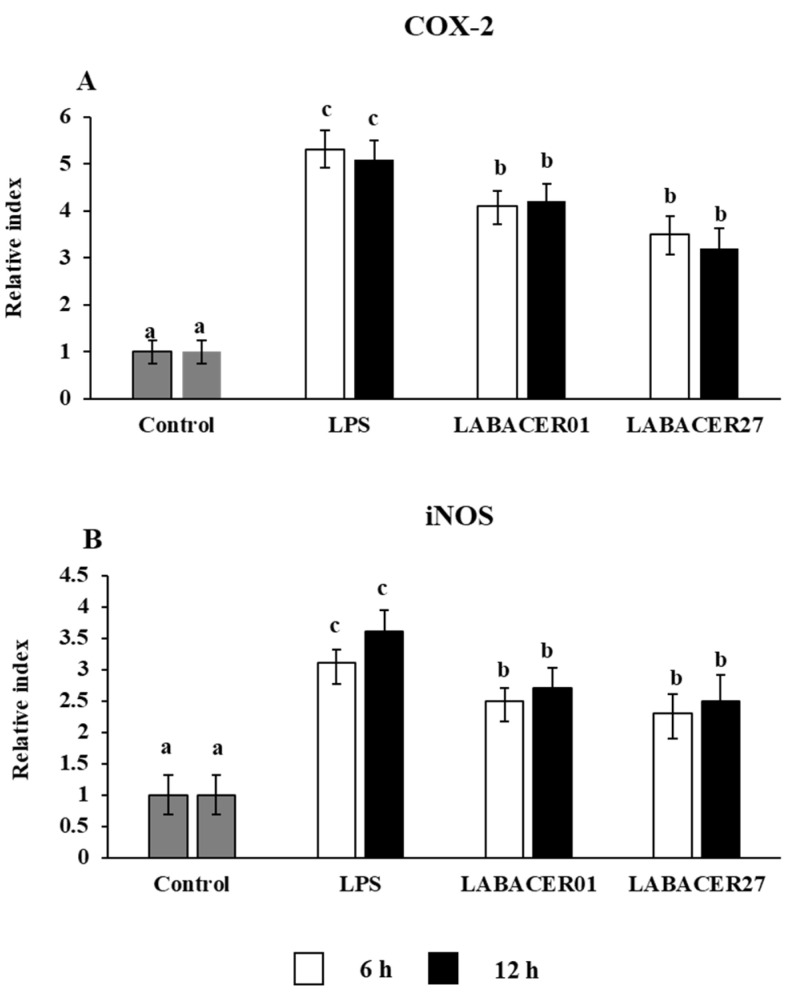
Relative expression of COX-2 (**A**), iNOS (**B**) in human colorectal epithelial Caco-2 cells challenged with KPC-2-producing hypermucoviscous ST25 strains of *Klebsiella pneumoniae*. Caco-2 cells were challenged with *K. pneumoniae* LABACER01, *K. pneumoniae* LABACER27, or LPS for 6 or 12 h. Untreated cells were used as controls. Results represent data from three independent experiments. Letters indicate significant differences between the groups, a < b < c, *p* < 0.05.

The expressions of the inducible nitric oxide synthase (iNOS) and cyclooxygenase 2 (COX-2) are upregulated by the intestinal epithelium after inflammatory challenges. Then, we evaluated the relative expression of iNOS and COX-2 in Caco-2 cells stimulated with LPS, LABACER01, and LABACER27. As shown in Figure 3, there was a significant upregulation in the relative gene expression of both COX-2 and iNOS in Caco-2 cells stimulated with LPS or challenged with the *K. pneumoniae* ST25 strains at 6 h and 12 h compared to unchallenged control cells. The expression levels for COX-2 and iNOS were similar for LABACER01 and LABACER27 strains, and both groups have lower levels of these genes compared to cells stimulated with LPS (Figure 3).

### 2.4. K. pneumoniae ST25 Trigger Inflammatory Factors Production in Caco-2 Cells

**Figure 4:**
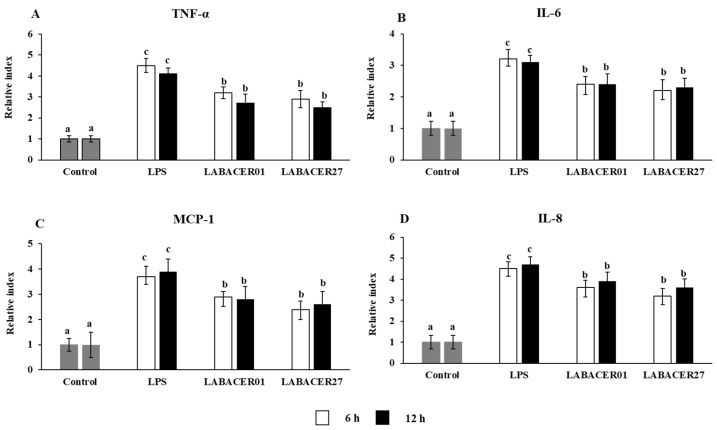
Relative gene expression of TNF-α (**A**), IL-6 (**B**), MCP-1 (**C**), and IL-8 (**D**) in human colorectal epithelial Caco-2 cells challenged with KPC-2-producing hypermucoviscous ST25 strains of *Klebsiella pneumoniae*. Caco-2 cells were challenged with *K. pneumoniae* LABACER01, *K. pneumoniae* LABACER27, or LPS for 6 or 12 h. Untreated cells were used as controls. Results represent data from three independent experiments. Letters indicate significant differences between the groups, a < b < c, *p* < 0.05.

**Figure 5:**
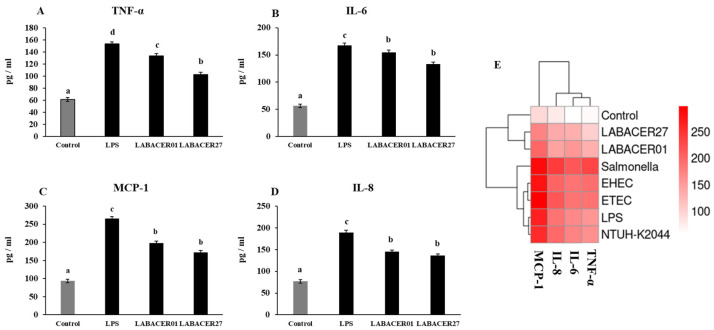
Concentration of TNF-α (**A**), IL-6 (**B**), MCP-1 (**C**), and IL-8 (**D**) in the supernatant of human colorectal epithelial Caco-2 cells challenged with KPC-2-producing hypermucoviscous ST25 strains of *Klebsiella pneumoniae* for 24 h. Caco-2 cells were challenged with *K. pneumoniae* LABACER01, *K. pneumoniae* LABACER27, or LPS. Untreated cells were used as controls. Results represent data from three independent experiments. Letters indicate significant differences between the groups, a < b < c, *p* < 0.05. (**E**) Heat map showing the concentration (pg/mL) of TNF-α, IL-6, MCP-1, and IL-8 in the supernatant of human colorectal epithelial Caco-2 cells challenged with KPC-2-producing hypermucoviscous ST25 strains of *K. pneumoniae* compared with Caco-2 cells challenged with *Salmonella* Typhimurium TUCO-I7 (Salmonella), enterohemorrhagic *E. coli* TUCO-I6 (EHEC), enterotoxigenic *E. coli* TUCO-I5 (ETEC), and *K. pneumoniae* NTUH-K2044 were used for comparisons. Cytokines were measured after 24 h of stimulation.

The relative expression of the inflammatory cytokines TNF-α, IL-6, MCP-1, and IL-8 in Caco-2 cells stimulated with LABACER01, LABACER27, or LPS were studied after 6 and 12 h (Figure 4). The challenge of Caco-2 cells with LPS or *K. pneumoniae* ST25 strains remarkably upregulated the expression of the four cytokines evaluated. However, cells treated with LPS had significantly higher levels of TNF-α, IL-6, MCP-1, and IL-8 than cells infected with LABACER01 or LABACER27 strains. In order to confirm the effect of *K. pneumoniae* ST25 in the production of inflammatory cytokines by Caco-2 cells, the concentration of TNF-α, IL-6, MCP-1, and IL-8 in culture supernatants were measured after 24 h of challenge (Figure 5). As expected, the group stimulated with LPS had higher levels of proinflammatory cytokines (TNF-α, IL-6) and chemokines (MCP-1, IL-8) compared to the cells challenged with *K. pneumoniae* ST25 strains. Both bacterial strains were equally effective in inducing the production of IL-6 (Figure 5B) and the chemokines MCP-1 and IL-8 (Figure 5C,D), while the LABACER01 induced significantly higher levels of TNF-α than the LABACER27 strain (Figure 5A). 

In order to compare the inflammatory capacity of the *K. pneumoniae* ST25 strains with other intestinal pathogens, Caco-2 cells were challenged with enterohemorrhagic *E. coli* TUCO-I6 (EHEC), enterotoxigenic *E. coli* TUCO-I5 (ETEC), *Salmonella* Typhimurium TUCO-I7, or *K. pneumoniae* NTUH-K2044, and the concentration of MCP-1, IL-8, IL-6, and TNF-α in culture supernatants were measured after 24 h (Figure 5E). The infection with *Salmonella* Typhimurium TUCO-I7 generated the highest inflammatory response, followed by the infection with ETEC and EHEC. Of note, the infection of Caco-2 cells with *K. pneumoniae* NTUH-K2044 strain induced levels of MCP-1, IL-8, IL-6, and TNF-α that were similar to those found in cells stimulated with LPS (Figure 5E). Both NTUH-K2044- and LPS-challenged cells had levels of inflammatory cytokines that were lower to cells infected with TUCO-I7, TUCO-I6, and TUCO-I5 strains. Interestingly, *K. pneumoniae* LABACER01 and LABACER27 induced changes in MCP-1, IL-8, IL-6, and TNF-α levels that were significantly lower than the other intestinal pathogens or LPS (Figure 5E).

### 2.5. K. pneumoniae ST25 Modulate TGF-β and TLL1 Expression in Caco-2 Cells

**Figure 6:**
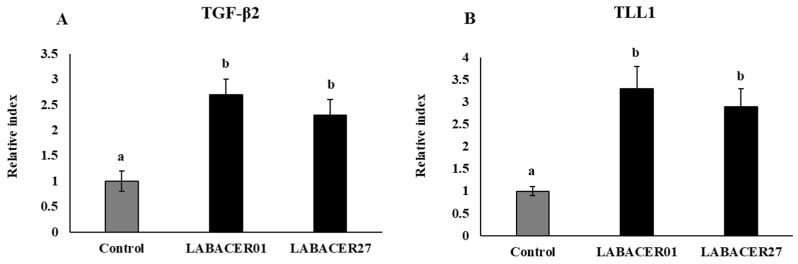
Relative gene expression of TGF-β2 (**A**) and TLL1 (**B**) in human colorectal epithelial Caco-2 cells challenged with KPC-2-producing hypermucoviscous ST25 strains of *Klebsiella pneumoniae*. Caco-2 cells were challenged with *K. pneumoniae* LABACER01, or *K. pneumoniae* LABACER27 for 12 h. Untreated cells were used as controls. Results represent data from three independent experiments. Letters indicate significant differences between the groups, a < b, *p* < 0.05.

Studies demonstrated that *K. pneumoniae* is able to modulate the TGF-β pathway to infect Caco-2 cells [19]. Next, we aimed to evaluate whether the *K. pneumoniae* ST25 strains modulated the expression of TGF-β2 and TLL1 in intestinal cells (Figure 6). Both LABACER01 and LABACER27 significantly increased the expression of TGF-β2 and TLL1 in Caco-2 cells compared to uninfected controls.

### 2.6. In Silico Analysis of Virulence Genes from K. pneumoniae ST25

In a previous study, a phylogenetic tree was constructed from 32 ribosomal protein subunit (rps)-coding genes present in several strains of *K. pneumoniae* [19]. In this phylogenetic tree, the strains LABACER01 and LABACER27, and the strains 28876 and SWU01 formed a separated phylogenomic group from other *K. pneumoniae* strains. Based on these previous results, we decided to carry out comparative studies of the virulence genes related to intestinal colonization/infection present in the genomes of these four strains. Additionally, we included the hypervirulent strain *K. pneumoniae* NTUH-K2044 for the genomic comparisons. An in silico analysis was performed to screen for the presence of crucial virulence factors associated with intestinal colonization/infection of *K. pneumoniae* as reviewed from the literature, including the following genes: colbactin [21,22,23,24], *magA* [9,10], *rmpA* [12,13], the TamA/TamB system [14], and the T6SS [15]. 

Of the strains studied, the highly virulent strain NTUH-K2044 was the one that presented the greatest number of virulence genes, being the only one in which the *magA* and *rmpA* genes were present (Table 1). All the strains presented the TamA/TamB system, and none presented the colbactin gene (Table 1). 

**Figure 7:**
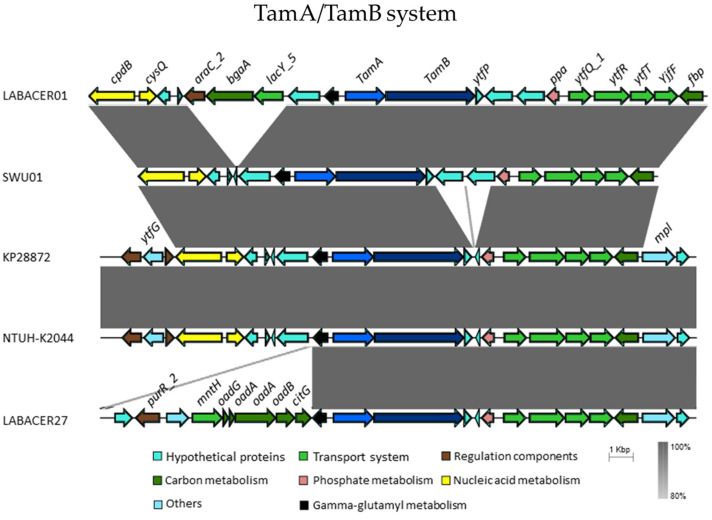
Synteny plot showing conservation around the Tam system genes in *K. pneumoniae* strains. The scale bar shows the level of nucleotide identity. The display has been obtained using EasyFig software 2.2.5 and the BLAST algorithm.

The synteny of the TamA/TamB system genes was found to be conserved in the analyzed strains, this system being found upstream of transport systems genes (*ytfQ_1*, *ytfR*, *ytfT*, and *ytfF*) and of fructose-bisphosphatase (*fbp*), and downstream of the genes *cpdB* and *cysQ* (Figure 7). 

**Figure 8:**
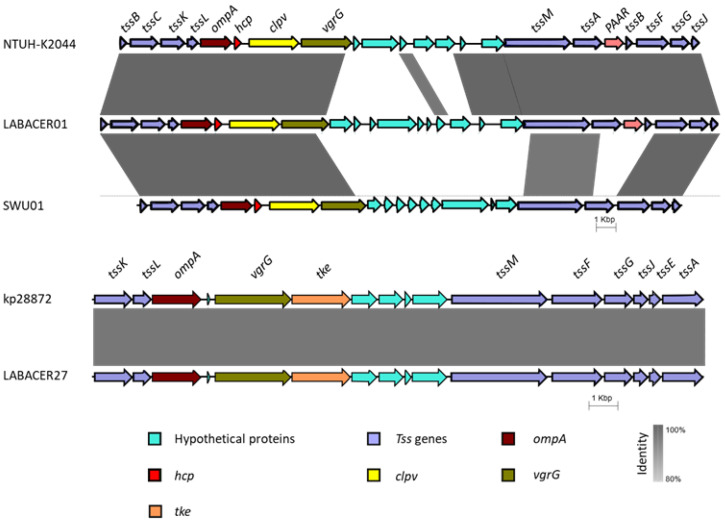
Synteny plot showing two different virulence clusters for Type VI Secretion System in *K. pneumoniae* strains. The scale bar shows the level of nucleotide identity. The display has been obtained using EasyFig software 2.2.5 and the BLAST algorithm.

In addition, at least one cluster for T6SS was identified in each strain. *K. pneumoniae* KP28872 and LABACER27 presented a similar cluster, composed of the genes: *tssA*, *tssE*, *tssF*, *tssG*, *tssI* (*vgrG*), *tssJ*, *tssk*, *tssL*, and *tssM* (Table 1, Figure 8). On the other hand, the LABACER01, NTUH-K2044, and SWU01 strains presented a cluster that differed from the previous one due to the presence of the *tssB*, *tssC*, *tssD* (*hcp*), and *PAAR* genes, and the absence of *tssE* (Table 1, Figure 8).

## 3. Discussion

Intestinal *K. pneumoniae* infections are relatively rare [8]; thus, there are few works describing the adhesion and invasion capacities of these bacteria to the intestinal mucosa. A study aimed to describe a possible mechanism for the gastrointestinal translocation of *K. pneumoniae* using Caco-2 cells cultures indicated that a Rho family GTPases and phosphatidylinositol 3-kinase (PI3K)/Akt signaling were necessary for bacterial translocation [16]. Another study evaluated the capacity of three clinically *K. pneumoniae* isolates to infect intestinal epithelial cells [8]. Interestingly, the strains had a different behavior when used to challenge Caco-2 cells being the strain KP1821, the one with the strongest invasive and adhesive abilities. In addition, in vivo experiments have demonstrated the ability of *K. pneumoniae* strains to colonize and infect the intestinal tract. Atarashi et al. [25] found that strains of *Klebsiella* spp. can induce a strong Th1 response when they colonize the gut of gnotobiotic mice. In IL-10^−/−^ gnotobiotic mice, it was found that *K. pneumoniae* 51-5 was capable of inducing the production of proinflammatory cytokines, DNA damage, and cell cycle arrest in the intestinal mucosa [26]. Additionally, in a co-colonization model with vancomycin-resistant enterococcus, using fluorescence in situ hybridization, high colonization levels of *K. pneumoniae* were found in the gastrointestinal tract of C57BL/6 mice [27]. Moreover, *K pneumoniae* strains were found to be able to invade colonic epithelial cells in a murine model [28]. To the best of our knowledge, the capacity of hypermucoviscous carbapenem-resistant *K. pneumoniae* with ST25 to infect and induce inflammation in the intestinal mucosa was not studied before.

In this work, we evaluated the adhesion and infection capacities of *K. pneumoniae* ST25 LABACER01 and LABACER27 in human intestinal epithelial Caco-2 cells. Both strains have different virulence patterns, as demonstrated by their distinct abilities to infect and damage the respiratory tract and disseminate into blood in mice when nasally administered [17,19,20]. However, no significant differences were found in the adhesion and invasion capabilities between both *K. pneumoniae* ST25 strains when used to challenge Caco-2 cells. In fact, the cell viability and LDH were similar in LABACER01 and LABACER27, indicating an equal capacity to produce damage to intestinal epithelial cells.

Previous studies showed the ability of *K. pneumoniae* to reduce tight junction-associated proteins claudin-1, ZO-1, and occludin [28]. However, studies demonstrated that *K. pneumoniae* is capable of translocating across a polarized Caco-2 monolayer without affecting transepithelial electrical resistance or altering tight junction protein ZO-1 or occludin distribution [16]. In line with those previous studies, we observed here that both *K. pneumoniae* ST25 strains did not alter the permeability of Caco-2 cells monolayer, although they reduced the expression of occludin, ZO-1, and claudin. Then, it is tempting to speculate that the ST25 strains, similar to other *K. pneumoniae* clones, can alter the expression of a tight junction without inducing obvious changes in protein expression since their translocation through the intestinal epithelium is via a transcellular pathway. On the other hand, the study of Lui et al. [8] demonstrated that the challenge of Caco-2 cells with clinical isolates of *K. pneumoniae* significantly increases the expression of the Tolloid-like TLL1, which is a protein that participates in several biological processes, including the TGF-β signaling pathways. Furthermore, the work reported that knocking down TLL1 in Caco-2 cells significantly diminishes the ability of *K. pneumoniae* to invade and adhere to the intestinal cells. Similar to this work, we observed here that *K. pneumoniae* ST25 strains significantly increased the expression of TGF-β2 and TLL1 in Caco-2 cells. These data indicate that ST25 strains can activate the TGF-β signaling pathway by increasing the expression of TLL1 to promote their adhesion and invasion in intestinal epithelial cells. Thus, our work demonstrates that ST25 strains would use mechanisms similar to those already established for other *K. pneumoniae* strains to adhere to and invade the intestinal epithelium.

Prostaglandins perform a key role in the inflammatory response, being their production associated with tissue inflammation. Previous studies in mice using TNBS-induced colitis showed that the oral administration of *K. pneumoniae* increased the expression of COX-2 [28]. In vitro studies using bovine retinal endothelial cells (BREC) found increased expression of cPLA2, iPLA2, COX-1, and COX-2 after *K. pneumoniae* stimulation. Transcriptomic studies revealed that the challenge of Caco-2 cells can up-regulate several inflammatory factors, including those related to the TNF signaling pathway [8]. It was also shown that *K. pneumoniae* strains can trigger inflammatory responses by inducing the production of proinflammatory cytokines. It was found that *K. pneumoniae* can multiply rapidly in bovine mammary epithelial cells cultures, inducing the expression of the proinflammatory cytokines TNF-α, IL-1β, IL-6, and IL-8, and the pattern recognition receptor TLR4, generating inflammatory damage and inducing apoptosis [29]. In addition, in supernatants of *K. pneumoniae*-stimulated cocultures of BREC and primary bovine retinal pericytes (BRPC), high levels of IL-6, IL-8, and vascular endothelial growth factor (VEGF) were found [30]. In line with these previous results, we demonstrated here that the challenge of Caco-2 cells with *K. pneumoniae* ST25 strains significantly increased the relative expression levels of COX-2 and iNOS, and at the same time, they reduced the expression levels of the tight junction-associated proteins claudin-1, ZO-1, and occludin. Furthermore, we demonstrated that the challenge of Caco-2 cells with LABACER01 and LABACER27 strains augmented the relative expression of inflammatory cytokines (TNF-α and IL-6) and chemokines (MCP-1 and IL-8). In agreement, increased production of these inflammatory factors was observed when determining their protein concentrations by ELISA.

Interestingly, both LABACER01 and LABACER27 strains were equally effective in augmenting the expression of COX-2, iNOS, TNF-α, IL-6, MCP-1, and IL-8, indicating a similar inflammatory potential in intestinal epithelial cells. In contrast, we demonstrated previously that these *K. pneumoniae* ST25 strains have a different inflammatory activity in the respiratory tract of immunocompetent adult mice [19]. Mice nasally infected with *K. pneumoniae* LABACER01 had higher levels of TNF-α, IL-1β, KC (IL-8 murine homologue), MCP-1, and IFN-γ in the respiratory tract and blood than animals challenged with LABACER27. In agreement, higher bacterial counts in the lungs were found for LABACER01 than LABACER27, showing that both *K. pneumoniae* ST25 strains are virulent and capable of infecting the respiratory tract and that the LABACER01 is more virulent than the LABACER27 strain [19]. Of note, the inflammatory response triggered by *K. pneumoniae* ST25 in Caco-2 cells was significantly lower than the ones induced by LPS stimulation or the challenges with other intestinal pathogens. Furthermore, the production of TNF-α, IL-6, MCP-1, and IL-8 by Caco-2 cells challenged with LABACER01 and LABACER27 were lower than the observed in cells infected with *K. pneumoniae* NTUH-K2044, which is a strain that efficiently colonizes and infect mice under experimental conditions [31,32]. It is tempting to speculate that the differences between LABACER01 and LABACER27 when comparing their virulence in the respiratory and intestinal tracts, and their differences with other pathogens can be explained by distinct expressions of virulence factors.

We previously performed a comparative genomic analysis that allowed us to define the groups of virulence factor genes present in the *K. pneumoniae* ST25 strains used in this work [19]. The results demonstrated that LABACER01 and LABACER27 possess virulence factors found in other hypervirulent *K. pneumoniae* strains. However, when analyzing virulence genes related to respiratory colonization, differences were found between LABACER01 and LABACER27. We speculated that these differences could explain the distinct magnitude of the activation of the respiratory and systemic inflammatory response observed in our in vivo studies [19]. According to this hypothesis, the virulence factors that are involved in the ability of the *K. pneumoniae* ST25 strains to colonize and infect epithelial cells should not differ among them. Then, we performed an in silico analysis to evaluate these factors in the genomes of LABACER01 and LABACER27.

The ability of *K. pneumoniae* strains to infect the intestine is conditioned by the repertoire of genes for colonization and competition. For instance, previous trials with *TamA*^−^ mutant strains demonstrated that this system is important for gut colonization [14]. The robust in silico analysis performed here demonstrated that the Tam system genes were found in both LABACER strains, although it was not previously detected in LABACER27 [19]. In contrast, both *K. pneumoniae* ST25 strains were *RmpA^−^*, *colbactin^−^*, and *MagA^−^*. The *RmpA* gene has been reported to be associated with liver abscess [33,34], whereas *MagA* was associated with clinical tissue-invasive diseases [35]. The NTUH-K2044 strain has the *RmpA* and *MagA* genes which are probably associated with the greater inflammatory effect observed in Caco-2 cells compared to LABACER strains. 

On the other hand, *Klebsiella* T6SS mediates antibacterial and antifungal competition [36]. All the strains analyzed in this work possess a T6SS cluster, considering those mentioned above with a minimum of 8 genes. However, it was found that *K. pneumoniae* strains have one of two types of T6SS clusters. *K. pneumoniae* LABACER01 has a T6SS cluster that is similar to the one found in the NTUH-K2044 strain, while LABACER27 and kp28872 strains possess a T6SS cluster similar to the previously reported in the strain CH1157-2 [37]. The T6SS components have been associated with differences in interbacterial and enterobacterial killing capacity [15,36]. The different T6SS clusters found in the two *K. pneumoniae* ST25 indicate that both would be equally effective in collaborating in the infection and inflammation induced in intestinal epithelial cells. It should be noted that the assembly level of LABACER01 and LABACER27 genomes is not complete, so the presence of other additional virulence clusters cannot be totally ruled out. Future research will focus on studying the expression of each of these T6SS genes, and quantifying the proteins through proteomics.

## 4. Materials and Methods

### 4.1. Strains

The strains LABACER01 and LABACER27, hypermucoviscous carbapenem-resistant *K. pneumoniae* ST25 strains, were isolated at the “Angel Cruz Padilla” hospital (San Miguel de Tucuman, Tucuman, Argentina). Both strains, were isolated from the intensive care unit and were selected based on their virulent capacity. *K. pneumoniae* ST25 strains were identified by matrix-assisted laser desorption/ionization (MALDI-TOF) (Microflex LT; Bruker Dal-tonik GmbH, Bremen, Germany) and kept in the Culture Collection of the Certified Bacteriology Laboratory (LABACER, National University of Tucuman, Tucuman, Argentina) [18]. *K. pneumoniae* LABACER01 and LABACER27 and the control strain NTUH-K2044 were grown in nutritive agar (Laboratorios Britania, Argentina) enriched with 5% defibrinated blood at 37 °C in 5% CO_2_ for 24 h. A single colony of each strain cultured on nutritive agar plates were inoculated into brain–heart infusion (BHI) broth and incubated at 37 °C for 18 h.

*Salmonella* Typhimurium TUCO-I7 (*Salmonella*), enterohemorrhagic *E. coli* TUCO-I6 (EHEC), and enterotoxigenic *E. coli* TUCO-I5 (ETEC) were provided by the Faculty of Veterinary Sciences, University of Concepción (Concepción, Chile). *E. coli* strains and *Salmonella* strains were grown in BHI broth at 37 °C for 18 h.

### 4.2. Cell Culture and Viability Assays

Human colorectal epithelial Caco-2 cells were maintained in Dulbecco’s Modified Eagle’s Medium (DMEM), supplemented with 20% heat-inactivated fetal bovine serum (FBS), and 1% non-essential amino acids (NEA) (all chemical substances from Gibco BRL, Carlsbad, CA, USA). The Caco-2 cells were cultured in tissue culture flasks in a CO_2_ incubator at 37 °C in a humidifier atmosphere and passaged by routine trypsinization. For experiments, Caco-2 cells were subcultured in DMEM supplemented with 10% FBS, 1% NEA, and 1% penicillin/streptomycin until they reached 80–90% confluence. The medium was changed once every two days., 

The MTT assay was used to determine the cell viability. In brief, Caco-2 cells were seeded at 1 × 10^4^ cells/well in a 96-well cell culture plate (Nunc, Thermofisher Scientific, Waltham, MA, USA). After 24 h incubation, cells were treated with hypervirulent *K. pneumoniae* ST25 LABACER01 and LABACER27 (10^3^ CFU) for 24 h. LPS (10 μg/mL) treatment was used for comparisons. Cells were incubated with MTT solution (2 mg/mL in serum-free medium, 50 µL/well) in each well for 4 h at 30 °C. Finally, the MTT medium was removed, and 150 µL DMSO was added to each well. The optical density was measured at 570 nm with an automated microplate reader (Bio-Rad, Hercules, CA, USA), and the data was expressed as the mean percentage of cell survival. Non-treated cells were considered as a control group.

### 4.3. Lactate Dehydrogenase (LDH) Activity Assay

The cellular damage induced by *K. pneumoniae* LABACER01 or LABACER27 was investigated by LDH release into the Caco-2 cell culture medium. The secretion of LDH was measured from the cellular supernatant using a commercially available LDH assay kit (Wiener-Lab, Buenos Aires, Argentina) according to the manufacturer’s instructions. The LDH assay is based on the conversion of lactate to pyruvate in the presence of LDH with parallel reduction in NAD to NADH. The change in the absorbance was recorded at 340 nm using an automatic biochemistry analyzer (Hitachi, Tokyo, Japan). The LDH activity was expressed as IU/l of supernatant.

### 4.4. Adhesion, Invasion, and Permeability Experiments in Caco-2 Cells

The adhesion and invasion capacity of *K. pneumoniae* LABACER01 or LABACER27 to Caco-2 cells were determined according to a previously described method by [8]. *K. pneumoniae* ST25 cells were used in the exponential growth phase. Bacteria were collected and resuspended in DMEM without fetal bovine serum (FBS).

For the adhesion assay, the human colorectal epithelial Caco-2 cells were seeded in a 24-well plate at a density of 10^5^ cells/well. Later, approximate 50 bacteria were used to infect each cell culture well and the cells were kept in an incubator for 2 h. After incubation, cells were washed three time with PBS, and 0.2% Triton X-100 was used to release the adherent bacteria. Adhesion was expressed as percentage of adhesion rate.

In the invasion experiment, Caco-2 cells were prepared as described above and challenged with *K. pneumoniae*. The cells were incubated for 2 h. Then, the medium was replaced with a medium containing colistin (10 µg/mL, Sigma-Aldrich, Buenos Aires, Argentina) and amikacin (5 µg/mL, Sigma-Aldrich) and incubated for 2 h to kill extracellular bacteria. Afterwards, the cells were washed three times with PBS, and 0.2% Triton X-100 was used to release the invading bacteria. The invasion of bacteria was expressed as 10^4^ CFU. Bacteria counts were determined in LB agar plates using gradient dilution. All experiments were repeated at least three times.

Permeability of Caco-2 cells monolayer was performed by the flux of FITC-dextran method. Caco-2 cells were seeded in a transwell chamber and FITC-dextran (1 mg/mL) (4 kDa, Sigma-Aldrich) was added to the apical compartment of the insets. After incubation of 6 h, the basolateral medium was collected for the measurement of fluorescence (480/520 nm excitation/emission).

### 4.5. RT-PCR

Caco-2 cells (10^5^ cells/well in a 24-well plate) were stimulated with *K. pneumoniae* LABACER01 or LABACER27 (10^4^ CFU/well) and the expression of several factors was evaluated at hour 6 and 12 post-stimulation. LPS (10 μg/mL) challenge was used as control for inflammation. Total RNA was isolated using TRIzol reagent based on the manufacturer’s instructions. In brief, cDNA was synthesized from 1000 ng of total RNA (MBI, Fermentas, St. Leon-Rot, Germany). The parameters of RT-PCR were 10 min 95 °C, then 95 °C for 15 s, and 60 °C for 1 min, performed for 40 cycles. The housekeeping gene was GAPDH. Primers used in this study are listed in Table 2. Primers for TGF-β2 and TLL1 were described previously [8].

### 4.6. Cytokine Concentrations

The Caco-2 cells (10^5^ cells/well in a 24-well plate) were stimulated with *K. pneumoniae* LABACER01, *K. pneumoniae* LABACER27, *K. pneumoniae* NTUH-K2044, enterohemorrhagic *E. coli* TUCO-I6 (EHEC), enterotoxigenic *E. coli* TUCO-I5 (ETEC), *Salmonella typhimurium* TUCO-I7 (10^4^ CFU/well), or LPS (10 μg/mL). Concentrations of cytokines were determined in Caco-2 cells supernatants after 24 h of stimulation. Control cells did not receive stimulation. TNF-α, IL-6, IL-8, and monocyte chemoattractant protein 1 (MCP-1) concentrations were measured with enzyme-linked immunosorbent assay (ELISA) kits following the manufacturer’s recommendations (R&D Systems, Minneapolis, MN, USA).

### 4.7. Genomic Analysis

The complete genome sequence of *K. pneumoniae* LABACER01 and LABACER27 were used to perform comparative genomic analysis focused on virulence factors. The strains and the characteristics of their genomes used in the study are detailed in Table 3. *K. pneumoniae* KP28872 (accession PRJNA630564), LABACER01 (accession PRJNA640366), LABACER27 (accession PRJNA640368), NTUH-K2044 (accession PRJDA21069), and SWU01 (accession PRJNA354663). Genomes downloaded from the Genbank were uploaded to the RAST server [38]. The search for virulence genes was carried out on this server using the BlastP algorithm. The synteny plots around the virulence genes were performed using the Easyfig v2.2.5 software [39] and the BLASTn algorithm.

### 4.8. Statistical Analysis

The experiments were performed in triplicate and the results were expressed as mean ± SD. Statistical analyses were performed using Prism 8.0 (GraphPad software, San Diego, CA, USA). Comparisons among multiple groups across multiple time points were performed using a two-way ANOVA with Tukey’s multiple comparison post hoc test. Comparisons between two groups were performed using unpaired Student’s *t*-tests. Differences were considered significant at *p* < 0.05.

## 5. Conclusions

This work demonstrated that *K. pneumoniae* ST25 strains were able to adhere and invade Caco-2 cells reducing their viability. Furthermore, both LABACER01 and LABACER27 strains reduced the expression of tight junction proteins (occludin, ZO-1, and claudin-5), altered permeability, enhanced the expression of TGF-β and TLL1, and increased the expression of inflammatory factors (COX-2, iNOS, MCP-1, IL-6, IL-8, and TNF-α) in Caco-2 cells. The inflammatory response induced by LABACER01 and LABACER27 was significantly lower than the produced by LPS stimulation or other intestinal pathogens challenges including *K. pneumoniae* NTUH-K2044. No differences in virulence and inflammatory potential were found between LABACER01 and LABACER27. In line with these findings, no major differences between the strains were found when the comparative genomic analysis of virulence factors associated with intestinal infection/colonization was performed. This work is the first in demonstrating that hypermucoviscous carbapenem-resistant *K. pneumoniae* ST25 infect human intestinal epithelial cells and induce moderate inflammation.

## Figures and Tables

**Table 1:** Virulence genes present in *K. pneumoniae* strains.

Strain	Colbactin	Mucoviscosity Associated Protein	RmpA	TamA/TamB System	Type VI Secretion System Main Cluster (Genes ≥ 8)
KP28872	-	-	-	+	*tssA*, *tssE*, *tssF*, *tssG*, *tssI* (*vgrG*), *tssJ*, *tssk*, *tssL*, *tssM*.
LABACER01	-	-	-	+	*tssA*, *tssB* (2 copies), *tssC*, *tssD* (*hcp*), *tssF*, *tssG*, *tssH* (*clpv*), *tssI* (*vgrG*), *tssJ*, *tssk*, *tssL*, *tssM*, *evpJ* (*PAAR*).
LABACER27	-	-	-	+	*tssA*, *tssE*, *tssF*, *tssG*, *tssI* (*vgrG*), *tssJ*, *tssk*, *tssL*, *tssM.*
NTUH-K2044	-	+	+	+	*tssA*, *tssB* (2 copies), *tssC*, *tssD* (*hcp*), *tssF*, *tssG*, *tssH* (*clpv*), *tssI* (*vgrG*), *tssJ*, *tssk*, *tssL*, *tssM*, *evpJ* (*PAAR*).
SWU01	-	-	-	+	*tssA*, *tssB*, *tssC*, *tssD* (*hcp*), *tssF*, *tssG*, *tssH* (*clpv*), *tssI* (*vgrG*), *tssJ*, *tssk*, *tssL*, *tssM*, *evpJ* (*PAAR*).

**Table 2:** Primer sequences used in this study.

Gene	Amplicon Size (pb)	Forward Primer (5′→3′)	Reverse Primer (5′→3′)
*Occludin*	114	GAGTTGTATCTGTTGTTGT	TTCGTGGTATAGCATTCT
*ZO-1*	118	GGTGAAGTGAAGACAATG	GGTAATATGGTGAAGTTAGAG
*Claudin-5*	84	TTAACAGACGGAATGAAGT	GAAGCGAAATCCTCAGTC
*COX-2*	117	GAGAGATGTATCCTCCCACAGTCA	GACCAGGCACCAGACCAAAG
*iNOS*	278	CCTTACGAGGCGAAGAAGGACAG	CAGTTTGAGAGAGGAGGCTCCG
*TNF-α*	360	GTCAGATCATCTTCTCGAACC	CAGATAGATGGGCTCATACC
*IL-6*	211	GACAGCCACTCACCTCTTCA	TTCACCAGGCAAGTCTCCTC
*MCP-1*	93	AGTCTCTGCCGCCCTTCT	GTGACTGGGGCATTGATTG
*IL-8*	168	ACTCCAAACCTTTCCACCC	CCCTCTTCAAAAACTTCTCCAC

**Table 3:** List of strains and genomes used in this study.

Strains	Assembly	Size	GC%	Scaffolds	CDS
KP28872	GCA_013623415.1	5.9	56.9	364	5553
LABACER01	GCA_013375175.1	5.6	57.1	61	5306
LABACER27	GCA_013375185.1	5.6	57.1	51	5170
NTUH-K2044	GCA_000009885.1	5.5	57.4	2	5028
SWU01	GCA_001902475.1	5.7	57.4	2	5420

## Data Availability

Data are contained within the article.
